# Same Day Discharge versus Overnight Stay in the Hospital following Percutaneous Coronary Intervention in Patients with Stable Coronary Artery Disease: A Systematic Review and Meta-Analysis of Randomized Controlled Trials

**DOI:** 10.1371/journal.pone.0169807

**Published:** 2017-01-09

**Authors:** Pravesh Kumar Bundhun, Mohammad Zafooruddin Sani Soogund, Wei-Qiang Huang

**Affiliations:** 1 Institute of Cardiovascular Diseases, the First Affiliated Hospital of Guangxi Medical University, Nanning, Guangxi, P. R. China; 2 Guangxi Medical University, Nanning, Guangxi, P. R. China; Humanitas Gavazzeni, ITALY

## Abstract

**Background:**

New research in interventional cardiology has shown the demand for percutaneous coronary interventions (PCI) to have increased tremendously. Effective treatment with a lower hospital cost has been the aim of several PCI capable centers. This study aimed to compare the adverse clinical outcomes associated with same day discharge versus overnight stay in the hospital following PCI in a population of randomized patients with stable coronary artery disease (CAD).

**Methods:**

The National Library of Medicine (MEDLINE/PubMed), the Cochrane Registry of Randomized Controlled Trials and EMBASE databases were searched (from March to June 2016) for randomized trials comparing same-day discharge versus overnight stay in the hospital following PCI. Main endpoints in this analysis included adverse cardiovascular outcomes observed during a 30-day period. Statistical analysis was carried out by the RevMan 5.3 software whereby odds ratios (OR) and 95% confidence intervals (CIs) were calculated with respect to a fixed or a random effects model.

**Results:**

Eight randomized trials with a total number of 3081 patients (1598 patients who were discharged on the same day and 1483 patients who stayed overnight in the hospital) were included. Results of this analysis showed that mortality, myocardial infarction (MI) and major adverse cardiac events (MACEs) were not significantly different between same day discharge versus overnight stay following PCI with OR: 0.22, 95% CI: 0.04–1.35; P = 0.10, OR: 0.68, 95% CI: 0.33–1.41; P = 0.30 and OR: 0.45, 95% CI: 0.20–1.02; P = 0.06 respectively. Blood transfusion and re-hospitalization were also not significantly different between these two groups with OR: 0.64, 95% CI: 0.13–3.21; P = 0.59 and OR: 1.53, 95% CI: 0.88–2.65; P = 0.13 respectively. Similarly, any adverse event, major bleeding and repeated revascularization were also not significantly different between these two groups of patients with stable CAD, with OR: 0.42, 95% CI: 0.05–3.97; P = 0.45, OR: 0.73, 95% CI: 0.15–3.54; P = 0.69 and OR: 0.67, 95% CI: 0.14–3.15; P = 0.61 respectively.

**Conclusion:**

In terms of adverse cardiovascular outcomes, same day discharge was neither superior nor inferior to overnight hospital stay following PCI in those patients with stable CAD. However, future research will have to emphasize on the long-term consequences.

## Introduction

Coronary artery disease (CAD) is one among the common chronic non-communicable diseases [1–2] affecting a large population of patients all around the world. New research in interventional cardiology has shown the demand for percutaneous coronary interventions (PCI) to have increased tremendously. Effective treatments with lower hospital costs have been the aim of several PCI capable centers. However, to further decrease their hospital expenses, patients are even willing to reduce their length of hospital stay following PCI. At the same time, interventionists and physicians are willing to provide an effective treatment along with a rapid hospital discharge following coronary angioplasty in order to arrange beds and provide space for new patients. Unfortunately, very few research has shown the impact of same day discharge versus overnight hospital stay in patients following PCI [3–6]. Therefore, this study aimed to compare the adverse clinical outcomes associated with same day discharge versus overnight stay in the hospital following PCI in a population of randomized patients with stable CAD.

## Methods

### Data sources and search strategy

The National Library of Medicine (MEDLINE/PubMed), the Cochrane Registry of Randomized Controlled Trials and the EMBASE databases were searched (from March to June 2016) for English language publications based on the comparison between same-day discharge versus overnight hospital stay following PCI by using the following terms:

1. Same day discharge, overnight stay and percutaneous coronary intervention;
2. Same day discharge and percutaneous coronary intervention;
3. Same day discharge, overnight stay, and coronary angioplasty;
4. Same day discharge, overnight stay and PCI;
5. Same day discharge, longer stay and PCI;
6. Outpatient and percutaneous coronary intervention/PCI;
7. Ambulatory and percutaneous coronary intervention/PCI;
8. SDD, OS and PCI;

These terms were also briefly searched in Google scholar and then the publications obtained were cross-checked in case we missed out any relevant article.

Reference lists of publications which were expected to be highly qualified for this analysis, and meta-analyses obtained during this search process were also checked for relevant trials.

This search included articles which were published between the years 1996 and 2016.

### Inclusion and exclusion criteria

Studies were included if:

1. They were Randomized controlled trials (RCTs) comparing same day discharge versus overnight hospital stay following PCI.
2. They consisted of patients with stable CAD.
3. They reported adverse clinical outcomes as their endpoints.

Studies were excluded if:

1. They were non-RCTs (meta-analyses, observational studies, case studies).
2. They did not compare same day discharge versus overnight hospital stay following PCI.
3. They did not involve patients with stable CAD.
4. They did not report any adverse clinical outcome following coronary angioplasty.
5. They were different studies but they were associated with the same trials and data.
6. They were duplicates, repeating themselves in different searched databases.

### Participants, outcomes and follow ups

#### Types of participants

This analysis mainly consisted of patients with stable CAD.

Outcomes analyzed included:

1. Death
2. Myocardial infarction (MI)
3. Major adverse cardiac events (MACEs) which consisted of death, MI and repeated revascularization. Since only one study reported Major adverse cerebrovascular and cardiovascular events (MACCEs), both MACCEs and MACEs were included in the same subgroup and analyzed.
4. Repeated revascularization
5. Major bleeding (any type of major bleeding)
6. Any adverse event
7. Re-hospitalization
8. Blood transfusion

The types of participants involved, the outcomes reported and the follow up periods have been summarized in Table 1. Most of the trials reported a follow up period of 30 days.

**Table 1:** Type of participants, outcomes and follow-up periods reported.

Trials	Type of participants	Outcomes reported	Follow up periods
**Knopf1999** [7]	Stable CAD	Any complication, MACEs	30 days
**Carere2000** [5]	Stable CAD and ACS	Any complication, MACEs	30 days
**Bertrand2008** [8]	Stable CAD and ACS	Death, MI, revascularization, major bleeding, transfusion, repeated hospitalization	30 days
**Clavijio2016** [9]	Stable CAD and ACS	MACEs, major bleeding, recurrent hospitalization, mortality	30 days
**Falcone2011** [10]	Stable CAD	Any adverse event	30 days
**Heyde2007** [11]	Stable CAD	MACCE, death, MI, stroke, revascularization	24 hours
**Kim2013** [12]	Stable CAD	MI, bleeding, hospitalization	30 days
**Slagboom2005** [13]	Stable CAD and unstable angina (only type A and B)	Death, MI, revascularization, adverse events, bleeding, transfusion	30 days

Abbreviations: MI: myocardial infarction, MACEs: major adverse cardiac events, MACCEs: major adverse cardiovascular and cerebrovascular events, CAD: coronary artery disease, ACS: acute coronary syndrome

### Data extraction and review

Two authors (PKB and MZSS) carefully reviewed the trials and retrieved data to be used in this analysis. The total number of patients who were discharged on the same day following PCI and those who stayed at least overnight in the hospital following PCI, the type of study reported (excluding observational cohorts), information regarding the baseline features of the patients, the clinical outcomes reported along with the respective follow up periods and the total number of events that occurred in both groups, were carefully extracted.

The bias risk was assessed in association with the Cochrane Collaboration [14]. Grades ranging from A (lowest risk of bias) to E (highest risk of bias) were allocated to each trial.

The PRISMA (Preferred Reporting Items for Systematic Reviews and Meta-Analyses) guideline was followed [15].

Any disagreement which came across during the data extraction process or the bias risk assessment was discussed and solved by the third author (WQH).

### Statistical analysis

Similar to many other meta-analyses, heterogeneity was expected in this analysis too. Heterogeneity would normally assess the null hypothesis in order to know if all the studies were assessing the same effect. In this analysis, first of all, heterogeneity was assessed using the Cochrane Q-statistic test and then secondly using the I^2^ statistic test.

A P value less or equal to 0.05 would denote a statistically significant result whereas a P value greater than 0.05 would imply an un-significant result.

I^2^ was required to measure inconsistency across subgroup analyses whereby a high percentage of I^2^ would denote an increased heterogeneity whereas a low I^2^ would signify a low level of heterogeneity.

A fixed effects model (I^2^ < 50%) or a random effects model (I^2^ > 50%) was used based on the value of I^2^ obtained during the data analysis.

RevMan software (version 5.3) was used to carry out the data analysis using odds ratios (OR) and 95% confidence intervals (CI) as the statistical parameters.

Publication bias was assessed through funnel plots.

Ethical approval was not considered necessary for this type of study.

## Results

### Database searched outcomes

A total number of 251 articles were searched from the electronic databases as well as from the reference lists of suitable meta-analyses and other relevant articles. After a careful review of the titles and abstracts, 214 articles were eliminated since they were not related to the scope of this analysis. A further 17 articles were eliminated since they were duplicates. Twenty (20) full-text articles were assessed for eligibility. Another selection was done whereby:

- two articles were eliminated since they were meta-analyses;
- three articles were eliminated since they were associated with the same trial;
- seven more articles were eliminated since they were observational studies.

**Fig 1:**
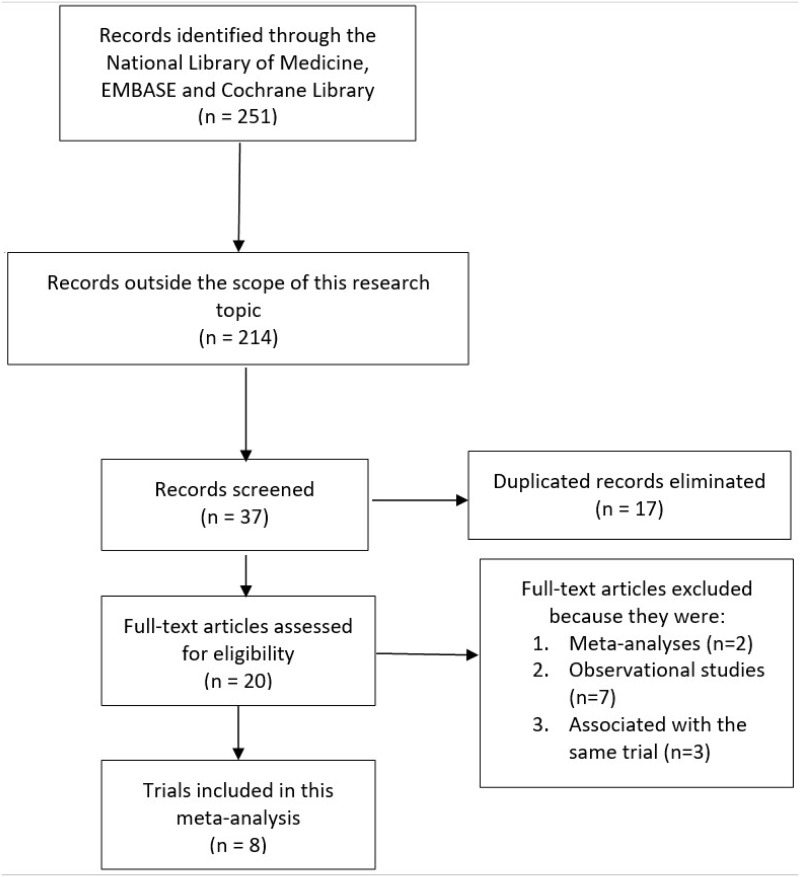
Flow diagram showing the study selection.

Finally, only eight trials satisfied all the inclusion and exclusion criteria of this analysis (Fig 1).

### General features of the trials

A total number of 3081 patients (1598 patients who were discharged on the same day and 1483 patients who stayed overnight in the hospital following coronary angioplasty) were included in this analysis. The most common features of the trials included in this meta-analysis have been listed in Table 2.

**Table 2:** General features of the trials.

Trials	Type of study	No of patients discharged on same day (n)	No of patients who stayed overnight (n)	Total number of patients (n)	Bias risk grade
**Knopf1999** [7]	RCT	43	47	90	B
**Bertrand2008** [8]	RCT	504	501	1005	B
**Clavijio2016** [9]	RCT	50	50	100	B
**Falcone2011** [10]	RCT	23	21	44	B
**Heyde2007** [11]	RCT	403	397	800	B
**Kim2013** [12]	RCT	150	148	298	B
**Slagboom2005** [13]	RCT	375	269	644	B
**Carere2000** [5]	RCT	50	50	100	B
**Total no of patients (n)**		1598	1483	3081	

Abbreviations: RCT: randomized controlled trials

### Baseline features of the patients

Table 3 summarizes the baseline features of the patients included in this analysis.

**Table 3:** Baseline features of the patients included.

Trials	Mean age*	Males (%)	Ht (%)	Ds (%)	Cs (%)	DM (%)
	SDD/OS	SDD/OS	SDD/OS	SDD/OS	SDD/OS	SDD/OS
**Knopf1999**	57.0/59.0	60.5/66.0	51.2/59.6	-	-	16.3/23.4
**Bertrand2008**	60.0/61.0	78.0/79.0	52.0/55.0	84.0/88.0	33.0/34.0	16.0/16.0
**Clavijio2016**	58.5/58.0	88.0/84.0	84.0/86.0	68.0/68.0	12.0/20.0	40.0/48.0
**Falcone2011**	60.6/57.0	65.2/85.7	86.9/95.2	86.9/95.2	30.4/28.6	47.8/42.9
**Heyde2007**	62.1/61.1	81.0/81.0	41.0/39.0	65.0/64.0	26.5/29.5	16.0/14.0
**Kim2013**	56.5/55.2	74.5/74.1	-	-	13.3/16.9	46.2/52.4
**Slagboom2005**	60.0/60.0	77.5/74.5	36.5/40.5	50.5/53.5	50.0/50.0	14.5/11.0
**Carere2000**	62.0/59.0	88.0/78.0	-	-	-	-

Abbreviations: SDD: same day discharge, OS: overnight stay, Ht: hypertension, Ds: dyslipidemia, Cs: current smoking, DM: diabetes mellitus;

*mean age was reported in years

According to Table 3, there was no significant difference in baseline features among patients who were discharged on the same day and patients who stayed overnight in the hospital following PCI.

### Adverse clinical and cardiovascular outcomes associated with patients who were discharged on the same day versus patients who stayed overnight in the hospital following coronary angioplasty

The main results have been summarized in Table 4.

**Table 4:** Results of this analysis.

Outcomes analyzed	OR with 95% CI	P value	I^2^ (%)
**Any adverse event**	0.42 [0.05–3.97]	0.45	71
**Death**	0.22 [0.04–1.35]	0.10	0
**Major bleeding**	0.73 [0.15–3.54]	0.69	72
**MI**	0.68 [0.33–1.41]	0.30	25
**MACEs**	0.45 [0.20–1.02]	0.06	0
**Blood transfusion**	0.64 [0.13–3.21]	0.59	0
**Repeated revascularization**	0.67 [0.14–3.15]	0.61	54
**Re-hospitalization**	1.53 [0.88–2.65]	0.13	0

Abbreviations: MI: myocardial infarction, MACEs: major adverse cardiac events, OR: odds ratio, CI: confidence intervals

**Fig 2:**
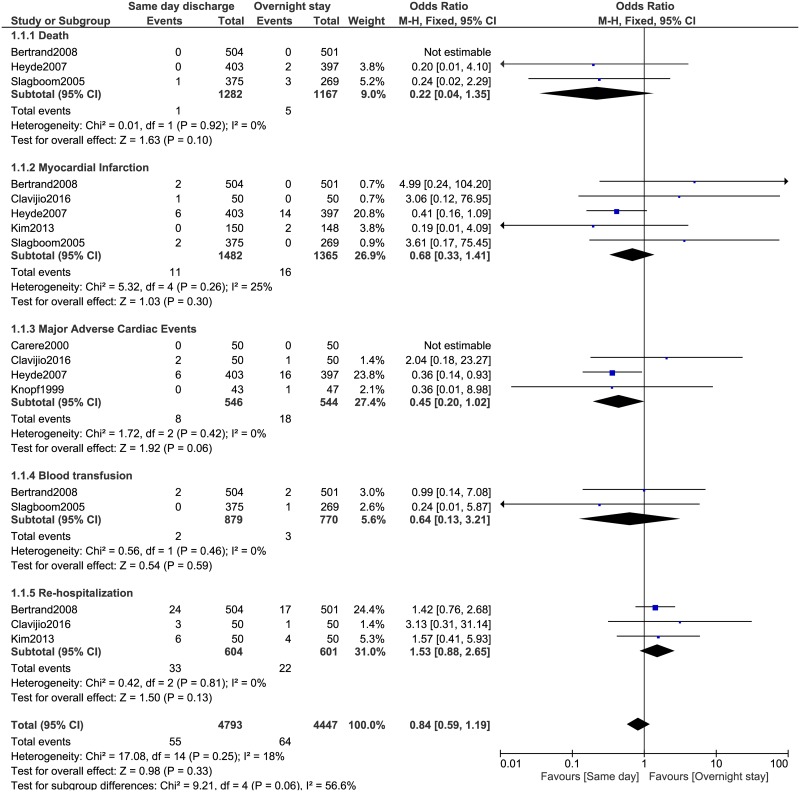
Adverse clinical outcomes associated with same day discharge versus overnight stay following coronary angioplasty (part 1).

During this 30-day follow up, mortality, MI and MACEs were not significantly different between same day discharge and overnight stay following PCI with OR: 0.22, 95% CI: 0.04–1.35; P = 0.10, OR: 0.68, 95% CI: 0.33–1.41; P = 0.30 and OR: 0.45, 95% CI: 0.20–1.02; P = 0.06 respectively. Similarly, blood transfusion and re-hospitalization were also not significantly different between these two groups with OR: 0.64, 95% CI: 0.13–3.21; P = 0.59 and OR: 1.53, 95% CI: 0.88–2.65; P = 0.13 respectively (Fig 2).

**Fig 3:**
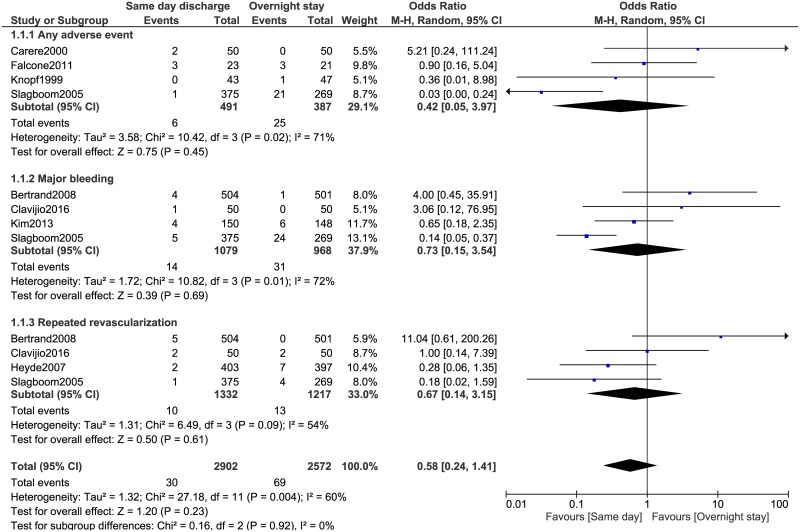
Adverse clinical outcomes associated with same day discharge versus overnight stay following coronary angioplasty (part 2).

Any adverse event, major bleeding and repeated revascularization were also not significantly different between these two groups with OR: 0.42, 95% CI: 0.05–3.97; P = 0.45, OR: 0.73, 95% CI: 0.15–3.54; P = 0.69 and OR: 0.67, 95% CI: 0.14–3.15; P = 0.61 respectively (Fig 3).

The subgroups analyzing adverse cardiovascular outcomes showed a low level of heterogeneity whereas a few other subgroups assessing other clinical outcomes showed moderate to high levels of heterogeneity.

**Fig 4:**
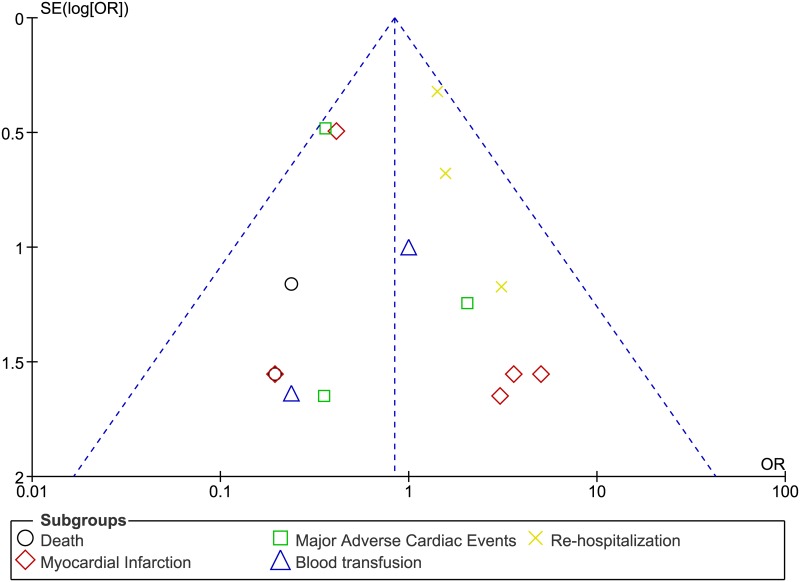
Funnel plot representing publication bias.

Fig 4 represents the funnel plot showing publication bias.

## Discussion

This systematic review and meta-analysis aimed to compare the adverse clinical outcomes associated with same day discharge versus overnight stay in the hospital following PCI. The current results showed that same day discharge was neither inferior nor superior to overnight hospital stay following coronary angioplasty in these patients with stable CAD, indicating that this rapid invasive procedure might later find a place in the outpatient setting especially for this particular subgroup of patients.

Similar to this analysis, Abdelaal et al [16] who evaluated the outcomes of same day discharge versus overnight hospitalization following PCI showed similar rate of MACEs associated with both discharge strategies and it should be noted that their analysis included a large number of patients (110,000 patients) obtained from observational studies and randomized trials. Brayton et al [17] also showed that in selected patients undergoing PCI, same day discharge was not associated with a higher rate of major adverse events and this strategy appeared to be as safe as the overnight following coronary angioplasty. They also showed that patients who were excluded from same day discharge following PCI had worse short and long term outcomes [18].

Similarly, an initial single centered experience [19] based on same day discharge following angioplasty showed this strategy to be safe and reliable in a selection of patients without resulting in complications thereafter. In addition, another single centered registry [20] demonstrated same day discharge to be safe in patients with low angiographic and clinical risks without showing any post-procedural complications.

In a study dealing with contemporary outpatient PCI [21], the authors also concluded that patients with single vessel or multi vessel PCI could be safely discharged within 10-hours post-procedure. Furthermore, the authors also suggested that outpatient PCI had the ability to reduce hospital cost and increase space for other patients who were waiting for their turn. This might also be the case even for high risk patients [22].

However, this was not always a correct decision. In a multi-centered cohort involving 107, 018 patients undergoing elective PCI between November 2004 and December 2008 [23], the authors concluded that among selected low risk patients, even if not associated with a high rate of mortality or re-hospitalization compared with overnight hospitalization, same day discharge should rarely be implemented in patients following PCI.

Nevertheless, with new advanced techniques in the catheter laboratories [24–25], even if same day discharge could find a place in PCI capable centers, concerns should be raised about the complications which might occur following this invasive procedure. In addition, close monitoring of the patients and proper explanations owing the long-term complications associated with PCI and their managements should also not be ignored in patients who require same day discharge following PCI. And it should be noted that without an overnight stay in hospital, monitoring and closely observing these patients for any complication would seldom be possible in such a short period of time especially for the subgroups of patients with acute coronary syndrome.

This interesting research idea should further be debated in clinical medicine since it is a question which is often asked by most of the patients who require PCI. This idea should also be made clear among interventionists in order to predict prognosis and to manage new patients in the outpatient settings in this fast-developing world.

### Novelty

This analysis might be considered new because it consisted of a large number of randomized patients. In addition to randomized patients, previously published meta-analyzes also involved patients obtained from observational studies which might have introduced several types of bias during subgroup analysis. Moreover, previously published meta-analyses did not include so many outcome subgroups. Associated with a low level of heterogeneity among the subgroups analyzing cardiovascular outcomes (mortality, MACEs) could be another new feature of this research.

### Limitations

A limited number of patients were analyzed and therefore, this analysis might not provide robust results. Also, the moderate and high levels of heterogeneity observed among several subgroups analyzing bleeding, any adverse event and repeated revascularization could be another limitation in this study. However, due to the fact that they were not the most important clinical outcomes in this analysis, they might not affect this analysis to a high extent. Selection bias, publication bias and language bias could have occurred because only English language publications were used in this analysis. Moreover, even if all the eight trials included in this analysis consisted of patients with stable CAD, three of the trials also included patients with low risk ACS, and one trial also included patients with unstable angina (type A and B). The inclusion of such patients along with stable CAD patients might have contributed to a heterogeneous result in several subgroups assessing other less important clinical outcomes. In addition, similar study design which was not reported in all of the eight trials might also have contributed to the introduction of bias across the studies.

### Conclusion

In terms of adverse cardiovascular outcomes, same day discharge was neither superior nor inferior to overnight hospital stay following PCI in those patients with stable CAD. However, future research will have to emphasize on the long-term consequences.

## Supporting Information

S1 PRISMA Checklist (DOC)

## Abbreviations

CAD: coronary artery diseases
MACEs: major adverse cardiac events
OR: odds ratio
PCI: percutaneous coronary intervention
RCTs: randomized controlled tri
